# Supplementary material for: Development of Ready-to-Eat Organic Protein Snack Bars: Assessment of Selected Changes of Physicochemical Quality Parameters and Antioxidant Activity Changes during Storage
Source: Foods. 2022 Nov 14;11(22):3631. doi: 10.3390/foods11223631 (PMC9689689; doi:10.3390/foods11223631)
Supplement: Supplementary file 1 [file foods-11-03631-s001.zip › foods-1945093-supplementary.pdf]

Table S1. Mean proportion of particular fatty acids (g/100g) and selected nutritional indices in investigated high protein bars (HPB) depending on the storage conditions

Item		Bar symbol								
		B1	B2	B3	B1	B2	B3	B1	B2	B3
		Before storage			After 3 months at 4°C			After 3 months at 22°C		
C 6:0	Caproic	0.10±0.05^{Aa}	0.10±0.05^{Aa}	0.10±0.05^{Aa}	0.10±0.05^{Aa}	0.10±0.05^{Aa}	0.10±0.05^{Aa}	0.10±0.05^{Aa}	-	-
C 8:0	Caprylic	1.40±0.07^{Bb}	0.23±0.10^{Ba}	0.27±0.10^{Ba}	1.07±0.05^{Ab}	0.17±0.10^{Aa}	0.17±0.10^{Aa}	1.23±0.06^{ABb}	0.27±0.10^{Ba}	0.27±0.10^{Ba}
C 10:0	Capric	1.20±0.06^{Bb}	0.27±0.10^{Bb}	0.30±0.10^{Bb}	0.90±0.10^{Ab}	0.17±0.10^{Aa}	0.17±0.10^{Aa}	1.03±0.05^{ABc}	0.30±0.10^{Aa}	0.30±0.10^{Bb}
C 12:0	Lauric	10.40±0.52^{Bc}	2.77±0.14^{Ba}	3.17±0.16^{Bb}	8,07±0,40^{Ab}	2.10±0.11^{Aa}	2.60±0.13^{Aa}	8.87±0.44^{Ab}	3.23±0.16 ^{Ca}	3.40±0.17^{BCab}
C 14:0	Myristic	4.27±0.21^{Cb}	1.37±0.07^{Ba}	1.47±0.07^{Bb}	3.33±0.17^{Aa}	1.07±0.05^{Aa}	1.20±0.06^{Ab}	3.67±0.1^{Ab}	1.37±0.07^{Ba}	1.47±0.07^{Bb}
C 15:0	Pentadecanoic	0.10±0.05^{Aa}	0.10±0.05^{Aa}	0.10±0.05^{Aa}	-	-	-	-	-	-
C 16:0	Palmitic	15.73±0.79^{Aa}	17.77±0.89^{Bb}	17.73±0.89^{Bb}	17.37±0.87^{Ba}	17.30±0.87^{Aa}	17.73±0.89^{Bb}	17.23±0.86^{Ab}	18.27±0.91^{Cc}	12.73±0.64^{Aa}
C 17:0	Heptadecanoic	0.10±0.05^{Aa}	0.20±0.13^{Ab}	0.20±0.10^{Ab}	0.20±0.10^{Ba}	0.20±0.10^{Aa}	0.20±0.10^{Aa}	0.13±0.10^{ABa}	0.20±0.10^{Aa}	6.10±0.31^{Bb}
C 18:0	Stearic	15.80±0.79^{Aa}	18.83±0.94^{Ab}	18.96±0.95^{Bb}	20.30±1.02^{Ca}	20.97±1.05^{Bb}	21.83±1.09^{Cc}	19.00±0.95^{Bb}	21.13±1.06^{Cc}	14.43±0.72^{Aa}
C 20:0	Arachidic	0.57±0.10^{Aa}	0.67±0.10^{Ab}	0.70±0.10^{Ab}	0.70±0.10^{Ba}	0.73±0.10^{Aa}	0.73±0.10^{Aa}	0.70±0.10^{Ba}	0.77±0.10^{Aa}	7.10±0.36^{Bb}
C 22:0	Behenic	0.13±0.10^{Aa}	0.13±0.10^{Aa}	0.20±0.10^{Aa}	0.20±0.10^{Aa}	0.20±0.10^{Aa}	0.20±0.10^{Aa}	0.13±0.05^{Aa}	0.20±0.10^{Aa}	0.37±0.10^{Bb}
C 24:0	Lignoceric	0.10±0.05^{Aa}	0.10±0.05^{Aa}	0.10±0.05^{Aa}	0.10±0.05^{Aa}	0.10±0.05^{Aa}	0.10±0.05^{Aa}	0.10±0.05^{Aa}	0.10±0.05^{Aa}	0.13±0.10^{Aa}
**Σ SFAs**		**49.90±2.50^{Ab}**	**42.50±2.13^{Aa}**	**43.26±2.16^{Ba}**	**52.33±2.62^{Ab}**	**43.03±2.15^{Aa}**	**44.93±2.25^{Ba}**	**52.20±2.61^{Ab}**	**45.83±2.29^{Ab}**	**31.23±1.56^{Aa}**
C 16:1	Palmitoleic	0.20±0.10^{Aa}	0.20±0.10^{Aa}	0.20±0.10^{Aa}	0.27±0.10^{Aa}	0.23±0.10^{Aa}	0.73±0.10^{Bb}	0.20±0.10^{Aa}	0.20±0.10^{Aa}	0.20±0.10^{Aa}
C 18:1cis9	Oleic	29.63±1.48^{Aa}	33.70±1.69^{Ab}	34.07±1.70^{Ab}	30.03±1.50^{Aa}	33.43±1.67^{Ab}	33.77±1.69^{Ab}	29.37±1.47^{Aa}	33.47±1.67^{Ab}	34.07±1.70^{Ab}
C 18:1cis11	Cis vaccenic	0.90±0.10^{ABa}	1.03±0.10^{Ba}	1.00±0.10^{Ba}	0.80±0.10^{Aa}	0.83±0.10^{Aa}	0.83±0.10^{Aa}	0.77±0.10^{Aa}	0.90±0.10^{Ab}	0.97±0.10^{Ab}

Σ C 18:1 other		-	-	-	-	0.30±0.10^{b}	0.10±0.05^{a}	-	-	-
Σ trans C 18:1		-	-	-	0.10±0.05^{Aa}	0.10±0.05^{Aa}	0.10±0.05^{Aa}	0.10±0.05^{Aa}	0.10±0.05^{Aa}	0.10±0.05^{Aa}
	Paullinic	0.20±0.10^{Aa}	0.27±0.10^{ABb}	0.30±0.10^{Ab}	0.20±0.10^{Aa}	0.23±0.10^{Aa}	0.27±0.10^{Aa}	0.20±0.10^{Aa}	0.30±0.05^{Ab}	0.30±0.05^{Ab}
**Σ MUFAs**		**30.93±1.55^{Aa}**	**35.20±1.76^{Aa}**	**35.57±1.78^{Aa}**	**31.10±1.56^{Aa}**	**34.50±1.73^{Aab}**	**35.33±1.77^{Ab}**	**30.33±1.52^{Aa}**	**34.57±1.73^{Ab}**	**35.23±1.76^{Ab}**
C 18:2 n6	Linoleic	17.60±0.88^{Ba}	20.67±1.03^{Bb}	19.27±0.96^{Bb}	14.97±0.75^{Aa}	20.40±1.02^{Ac}	17.67±0.88^{Ab}	15.87±0.79^{Aa}	17.60±0.88^{Abc}	16.87±0.84 ^{Ab}
Σtrans C 18:3		-	0.10±0.05^{Aa}	0.10±0.07^{Aa}	0.20±0.10^{Aa}	0.23±0.10^{Ba}	0.27±0.10^{Ba}	0.20±0.10^{Aa}	0.30±0.05^{Bb}	0.30±0.05^{Bb}
C 18:3 n3	α- linoleic (ALA)	1.57±0.08^{Ba}	1.60±0.08^{Bb}	1.80±0.09^{Bbc}	1.30±0.07^{Aa}	1.40±0.07^{Aab}	1.47±0.07^{Ab}	1.23±0.06^{Aa}	1.53±0.08^{Bb}	1.63±0.08^{ABb}
**Σ PUFAs**		**19.17±0.96^{Ba}**	**22.30±1.12^{Bb}**	**21.17±1.06^{Bb}**	**16.27±0.81^{Aa}**	**21.08±1.09^{Ac}**	**19.13±0.96^{Ab}**	**17.01±0.86^{Aa}**	**19.13±0.96^{Ab}**	**18.50±0.93^{Ab}**
**PUFA/SFA[1]**		0.38	0.52	0.48	0.31	0.49	0.42	0.32	0.42	0.59
**(IA)[2]**		0.98	0.73	0.76	1.10	0.78	0.83	1.10	0.85	0.58
**C18:2/C16:0[3]**		1.12	1.18	1.09	0.86	1.16	1.03	0.92	0.96	1.32

Explanatory notes:
Table shows mean values ± standard deviations.
Values denoted by different capital letters in the same batch of bars, at different temperature storage, differ significantly ($p < 0.05$). Values denoted by different lowercase letters in the same conditions of storage, differ significantly ($p < 0.05$).
Refer to **Table 4** for identification of test samples.
MUFAs - monounsaturated fatty acids ; PUFAs - polyunsaturated fatty acids ; SFAs – saturated fatty acids, TFAs- trans fatty acids; „-"wasn't detected.
[1] PUFA/SFA, the ratio of polyunsaturated fatty acid to saturated fatty acid,
[2] IA - Index of Atherogenicity.
[3] C18:2/C16:0 - the ratio of linoleic acid to palmitic acid
